# Colour as a behavioural guide for fish near hydrokinetic turbines

**DOI:** 10.1016/j.heliyon.2023.e22376

**Published:** 2023-11-17

**Authors:** Guglielmo Sonnino Sorisio, Stephanie Müller, Catherine A.M.E. Wilson, Pablo Ouro, Jo Cable

**Affiliations:** a School of Engineering, Cardiff University, CF24 3AA, UK; b School of Engineering, University of Manchester, M13 9PL, UK; c School of Biosciences, Cardiff University, CF10 3AX, UK

**Keywords:** Turbine, Fish, Passage, Colour, Trout, Salmonid

## Abstract

Hydropower is a traditional and widespread form of renewable energy and vertical axis turbines are an emerging technology suitable for low to medium velocity water bodies such as rivers. Such devices can provide renewable power to remote communities but may also contribute to fragmenting already poorly connected riverine habitats and the impact could be particularly pronounced for migratory diadromous aquatic species such as salmonids by limiting their ability to pass the turbines. Optimising the design of such turbines is therefore essential to mitigate their impact on aquatic fauna. One easily altered property that does not impact turbine performance is blade colour. Here, juvenile rainbow trout (*Oncorhynchus mykiss*) free swimming within a flume were monitored in the presence of a vertical axis turbine that was either stationary or rotating, and coloured white or orange. The orange colour of the turbine affected behaviour by increasing turbine avoidance and decreasing the number of potentially harmful interactions with the turbine when it was rotating, whilst not affecting passage or mobility of the trout compared to the white turbine. Visibility is therefore a potentially useful tool in mitigating the environmental impact of hydrokinetic turbines.

## Highlights

• Rainbow trout were exposed to vertical axis turbines of different colours in the laboratory.
• Passage, behaviour, and trajectories around the turbine were observed.
• Interactions with a spinning turbine decreased when painted orange.
• There was no decrease in passage as a result of colour change.

## Graphical abstract

**Figure:**
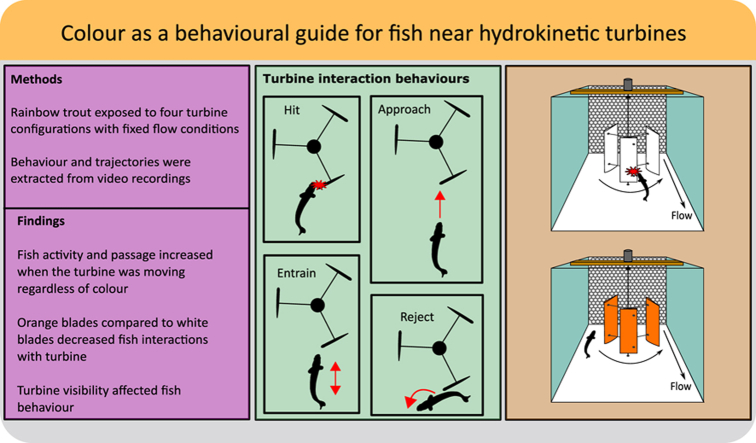

## 1 Introduction

Renewable energy is becoming more widespread both in number and type of installation to meet global targets. This is especially apparent with recent volatility in fossil fuel prices and the necessity to reduce national dependance on fossil fuel imports [1]. Taking the European Union directives as an example, current targets from the 2018 recast directive are 32 % share of renewables by 2030 but current proposals under REPower EU aim to increase this to at least 45 %, a large increase from the 2020s 22 % renewable energy share [1,2]. Hydropower is an established technology of renewable energy but a major drawback of large hydropower schemes that span the width of the river is that they fragment the river course, separating habitats and migration pathways of diadromous fish species that move between the sea and freshwater [3,4]. Upstream migrating fish are often delayed or prevented from travelling upstream of large dams even when passage is provided [5] and downstream migrating fish may encounter extremely high mortality rates, injuries, or delays [6]. Dams also impound water and prevent sediment transport in rivers [7,8], which can be detrimental to habitats downstream.

An emerging form of renewable energy schemes that partially mitigates the problem of fish passage is the hydrokinetic turbine. Vertical axis turbines (VAT) are particularly suitable to riverine applications, these devices do not require large alterations to the flow of the river, do not impound water and do not block the entire width of the river. Therefore, as an alternative means of producing renewable energy, they may have a reduced effect on fish migration and allow the river to remain, at least partially, connected. VATs are a suitable solution to produce power in remote communities out of reach of the main grid because of their low cost, ease of installation and wide range of operating conditions [9]. VATs have been deployed in remote areas all over the globe and across all continents and applied to a diverse range of water bodies, from irrigation channels to large river systems [[10], [11], [12], [13]]. Little is known, however, about their impact on fish movement and welfare, posing the question whether VATs present a migration barrier. Like other salmonids, rainbow trout (*Oncorhynchus mykiss*) are important to wild fisheries and angling communities, having a global monetary value of up to €500 million annually [14], in addition to high conservational value. Most salmonids are anadromous, they migrate from freshwater to the sea as juveniles (known as smolts) and return to freshwater as adults. These migrations are essential for completing their life cycle but are often disturbed by anthropogenic barriers such as hydropower schemes. Rainbow trout, having a high trophic role, have highly developed eyesight, relying on it to navigate through turbulent flows, for hunting, and are able to perceive ultraviolet and polarized light [[15], [16], [17], [18], [19]]. The auditory capabilities of trout are of importance when a device might also generate sound, although, rainbow trout do not appear to be sensitive to sounds produced by hydrokinetic turbines [20,21]. There is not enough data on the effect of VATs on fish passage and potential blade strike risk, but we know VATs influence the flow by creating areas of low velocity flow in their wake with high turbulent intensity and kinetic energy [22,23]. In an *in situ* study where the VAT was small in comparison to the river width, brown trout (*Salmo trutta*) avoided the turbine at all times while Atlantic salmon (*Salmo salar*) only avoided it when rotating [24]. In marine settings and in lab experiments, fish displayed reduced movement around VATs [25,26]. Encouragingly, studies have found that no juvenile Atlantic salmon nor brown trout contacted a VAT under experimental conditions and that time spent in the vicinity of the turbine did not lead to increased mortality or injury in Atlantic salmon and American shad [27]. However, the different species behave differently, salmon are generally bolder than brown trout and shad. With increasing flow velocity salmon and trout passed through the turbine more often [27]. Fish species is therefore a factor that affects passage, but turbine placement and the blockage of the turbine can also affect how the fish interact with the turbines [28].

Rainbow trout are native to freshwater catchments connected to the Pacific Ocean from North America and Northern Russia to Mexico [29] but they have been introduced to many other countries where VATs are likely to be installed to support renewable energy production. Therefore, rainbow trout have been chosen as a model species to assess the impact of such devices. Instream VATs can limit rainbow trout movement as measured in a laboratory flume [28] but changing features of their design might help to reduce their environmental impact. One of the simplest alterations to wind turbines, which are typically white, is to colour one of the blades black, reducing the number of bird fatalities by 70 % [30], indicating visibility could be key to preventing high strike rates. In the presence of a natural flood barrier, colour significantly affected fish passage with orange leading to increased passage compared to the natural wood colour of the barrier [31]. Colour has also been used in the form of strobes to deter fish, as well as being used as a guidance technique [[32], [33], [34], [35], [36]]. The strobes most often cause negative phototaxis (movement away from light) and the effectiveness of this solution can change with either strobe rate or colour of the light emitted [32]. Turbine visibility has been identified as a knowledge gap [37], consequently, colour might be a non-invasive and inexpensive solution to mitigate potential negative impacts of turbines on multiple species, which serves as the motivation for this study.

In this study, we assess whether turbine colour can affect fish passage and reduce collision risk by evaluating the effect of VAT blade colour on the behavioural response of rainbow trout. Passage statistics, spatial and hydrodynamic preferences and the reactions of the fish were assessed in an open-channel flume with two differently coloured turbines (a white and an orange) with either rotating or stationary blades under the same bulk velocity and flow conditions.

## 2 Methods

2.1 Animal source, maintenance, and experimental set-up.

Rainbow trout (*Oncorhynchus mykiss*) (N = 80) sourced from Bibury Trout Farm, Gloucestershire, (UK) were transported to and maintained in the Aquatics laboratory of Cardiff School of Biosciences in a Recirculating Aquaculture System (RAS). Trout were fed daily with Skretting pellets. Shortly before the experiment, the trout were moved to a circular tank (diameter = 1.3 m, height = 0.6 m) filled with dechlorinated water at 13.5 ± 0.5 °C with a 10h:14h light:dark cycle. Water from the main tank was filtered (Aquamanta, EFX 600 External Canister Filter), cooled (D-D Aquarium Solution, DC 750) and then pumped into a sump tank before being pumped back into the main tank. The tank was covered with a net to prevent trout from jumping out while still allowing light into the tank. Aeration was provided by air pumps and tank enrichment consisted of stones, pipe sections and ceramic pots. The trout were kept in this tank at a density of 2 L per fish for at least one week prior to the flume experiments. There was no significant difference between treatments in fish standard length, total length, and mass (GLM, *p* > 0.05), the average values for these measurements are given in Table 1.

**Table 1:** Average lengths (and standard deviation) of the fish used in all four treatment groups, with 20 fish tested per treatment.

Treatment Name	Standard Length (mm)	Total Length (mm)	Mass (g)
White Rotating (WR)	56.2 ± 5.2	65.7 ± 5.7	2.9 ± 0.7
White Stationary (WS)	57.0 ± 5.5	67.9 ± 7.6	3.4 ± 0.9
Orange Rotating (OR)	56.1 ± 5.8	67.4 ± 6.3	2.8 ± 0.9
Orange Stationary (OS)	56.3 ± 7.3	66.8 ± 7.5	2.9 ± 1.0

**Fig. 1:**
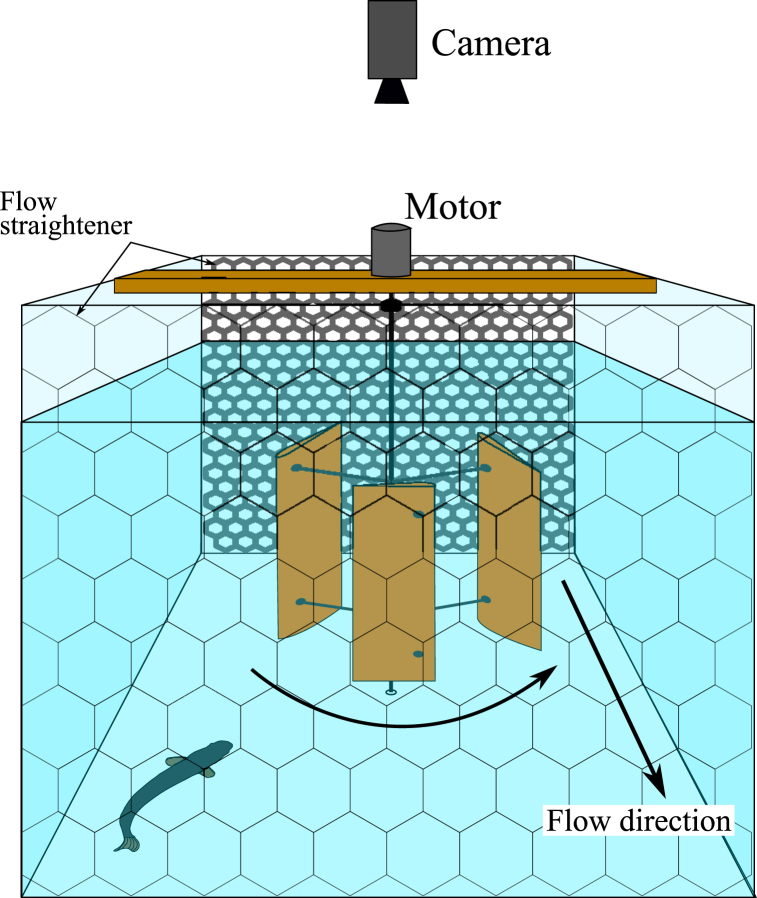
Experimental setup of the working area through the flume cross-section, looking in the upstream direction. The motor that drives the vertical axis turbine (VAT) is mounted on a support structure that holds the turbine vertically and the bottom of the turbine shaft is inserted into a bearing on the flume bed. The sides of the area are bounded by glass walls, and the upstream and downstream ends bounded by flow straighteners. The camera is mounted above the flume to record fish behaviour.

The open channel recirculating flume located at Cardiff School of Engineering (UK) used for this study was 10 m long, 0.3 m wide and 0.3 m tall with a fixed 1/1000 bed slope and glass side walls. The 1.2 m long working section was located at 4.8 m–6 m from the upstream inlet of the flume, bounded by aluminium honeycomb flow straighteners (metallic grey). At the water surface a perspex sheet was used to enhance the view from above. The vertical axis turbine, illustrated in Fig. 1, was mounted on the center line of the working section. The flume bed was made from white plastic. The flume was filled with dechlorinated water and the temperature controlled (D-D Aquarium Solution, DC 2200) and maintained at 15 ± 2 °C. After the experiments, the trout were transferred to a secondary holding tank with similar environmental conditions adjacent to the flume. At the end of the experimental day, the trout were transported back to the Aquatics lab and not re-used.

The turbine used in this experiment was a vertical axis turbine (Fig. 1) with a diameter and height of 120 mm, representing an 18:100 scale resolution. The turbine comprised of a 6 mm diameter central shaft to which three blades were mounted [22]. Each blade had a NACA0015 profile and chord length of 30 mm, and was mounted to the shaft using two 3 mm diameter struts. The blades were additively manufactured using laser sintering with PA2200 nylon. The central shaft and the struts were stainless steel, which held the bottom of the blades 20 mm above the flume bed. The geometric scale of the turbine diameter in relation to the mean standard fish length was approximately 1:2.15.

The bottom end of the turbine's central shaft is inserted into a bearing in the flume bed to allow it to rotate freely while the upper end was connected to the motor and encoder which were mounted on a plastic beam, spanning the width of the flume to hold the turbine in place. The turbine was located centrally within the working section, 0.6 m from each flow straightener and 0.15 m from each sidewall. The colour of the blades when manufactured was white and this represented our control treatments. For two of our four treatments, the blades were painted orange with custom orange paint mixed to be equivalent to 610 nm, this was chosen to match a range of red colours that affected fish behaviour in previous studies. The water-resistant paint was applied in two coats, it was left to dry completely before being mounted onto the turbine body, and it did not degrade over the test period.

The working area of the flume was lit by four neutral white lights with average illuminance of this area being 1858 lx (measured with a Testo 540 Pocket Light Meter at 10 locations across the working area and spatially averaged). The fish behavioural trials were recorded by two cameras, a Baumer camera (Baumer VLXT-50 M.I) mounted above the flume recording at 80 frames per second (fps) in greyscale, and a GoPro Hero 5 camera on the side of the flume recording through the glass wall at 25 fps in colour.

### 2.1 Experimental design

Flow depth and bulk velocity were kept constant over all treatments and along the length of the flume, the flowrate (Q) was 13 L/s and the flow depth (h) was adjusted to be 0.23 m by the weir at the downstream end of the flume, producing a bulk velocity ($\bar{U}$) of 0.19 m/s. These conditions correspond to a flow Reynolds number based on the hydraulic radius of the flume (R) $Re=\frac{\rho \bar{U}R}{\mu }$ of 13,184 where ρ is the fluid's density and μ is the dynamic viscosity. Four treatment conditions were tested: WS, WR, OS and OR, where W = white blades, O = orange blades, S = stationary turbine and R = rotating turbine, outlined in Table 2. When the turbine was rotating, the rotational speed (ω) was set to 59 rotations per minute (RPM). For each of the four experimental treatments, N = 20 fish were used per treatment with a total of 80 fish used. The bulk velocity was chosen to be in the range of the sustained swimming speed for the fish to avoid fish becoming exhausted which would affect the results [38].

**Table 2:** Treatment details and flow conditions. Flowrate (Q) and flow depth (h) were kept constant whilst the turbine speed (ω) and the turbine colour varied.

Treatment Name	$\bar{U}$ (ms^−1^)	h (m)	Q (Ls^−1^)	ω (rpm)	Turbine Colour
White Rotating (WR)	0.19	0.23	13	0	White
White Stationary (WS)	0.19	0.23	13	59	White
Orange Rotating (OR)	0.19	0.23	13	0	Orange
Orange Stationary (OS)	0.19	0.23	13	59	Orange

The fish were allowed to acclimate to flume conditions for 20 min prior to 10 min of behavioural recording. During acclimation, flow velocity was increased in three steps and if the turbine was rotating then its speed was also increased in three steps. Before each fish were released into the working area by net, the flowrate was adjusted to 6 L/s and the turbine to 7 RPM. After 5 min, the flowrate was increased to 9 L/s and the turbine to 30 RPM, after a further 5 min the flowrate was increased again to 13 L/s and the turbine to 59 RPM. To complete acclimation, each fish was left for a further 10 min before it was removed from the flume and placed in a temporary tank producing a total acclimation time of 20 min per fish followed by the test. The working area was then covered with a 10 mm thick plexiglass sheet to avoid reflections of the water surface when the image was captured by the camera above the flume. The camera was set to record for 10 min and 30 s, with the first 30 s accounting for the time in which the trout were re-released at the downstream end of the working area. On completion of the flume trial, the plexiglass sheet was removed, the trout re-captured and the standard length, total length and mass measured with vernier calipers and scales. The total time each trout was in the flume for was therefore 30 min and 30 s; 20 min of acclimation and 10.5 min of test.

### 2.2 Data analysis

**Fig. 2:**
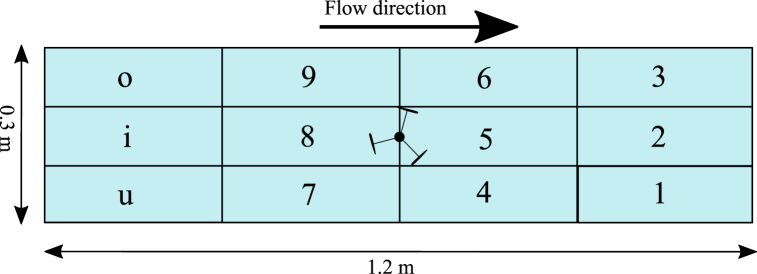
The working area represented as smaller subsections and the keyboard codes used to pair the location of behaviours (Table 3) with location within the working area. The letters U, I and O were used to denominate the furthest upstream zones. Turbine not to scale.

To analyse the behaviour of the trout under the four different treatments, video recordings were analysed using JWatcher software v1.0 [39,40]. This software allows the user to specify behaviours and assign keys to them so that when a particular behaviour starts and the associated key is pressed, the software will record the time (±1 ms) and duration of this behaviour. Duration was calculated by recording the amount of time elapsed until the next key was pressed as behaviours (Table 3) are assumed to be mutually exclusive unless specified otherwise. Modifiers can be added to the behaviours and each modifier is also assigned a key; for this study, adding modifiers allowed further differentiation between swim forward, swim backward, station holding, and passing behaviours. The working area was split into 12 sections as shown in Fig. 2 and each section was assigned a corresponding key to work as a modifier for these four behaviours. JWatcher includes an analysis function which processes the raw behavioural data and presents a summary for each behaviour and modifier for both the total time the fish spent in each behaviour and the number of total times the behaviour was observed.

**Table 3:** Behaviours and their descriptors used to analyse the video data at a reduced framerate of 40 fps. Further clarification of the modifiers is given in bold, see Fig. 2.

Behaviour	Description
Swim Forwards	The fish swims forward more than one fish length while facing upstream (positive rheotaxis); **subject to modifiers in**Fig. 2**.**
Swim Backwards	The fish swims, either facing upstream (drifting) or downstream (negative rheotaxis), more than one fish length in the downstream direction; **subject to modifiers in**Fig. 2**.**
Station Holding	The fish swims steadily and remains in the same place± 2 BL; **subject to modifiers in**Fig. 2**.**
Rest	The fish does not swim and rests either against the downstream flow straightener, wall or on the flume bed.
Approach	The fish swims directly upstream towards the turbine and reaches within one turbine diameter of the turbine.
Entrain	The fish swims in the near wake of the turbine (within 2 turbine diameters) and holds station in the near wake.
Pass	The fish passes by or through the turbine and moves from either the upstream area into the downstream area or from the downstream area into the upstream area. **Modifiers Y and D were used to differentiate between up and downstream passes.**
Rejection	The fish approaches the turbine or attempts to pass by but sharply turns and quickly swims away.
Far Wake Swimming	The fish swims in the wake of the turbine and holds station more than 2 turbine diameters downstream of the turbine.
Front	The fish swims within 1 turbine diameter upstream of the turbine and holds station facing upstream, also known as bow-waking.
Hit or Strike	The fish makes physical contact with the turbine blade.

Reliability testing was carried out for JWatcher to assess scoring accuracy. Three videos were randomly sampled from the dataset and analysed twice, one week apart. The reliability test function was used to estimate accuracy which was evaluated to be 94.3 % (mean average of the accuracy for three videos). The main sources of error came from differences in key order and timing when the fish moved quickly. These differences had a negligible overall impact on the video analysis. Time spent resting was not included in the analysis of time spent in each section of the flume as the only place the fish could rest was against the downstream straightener or on the flume bed immediately upstream of it. This would lead to the downstream sections of the working area appearing to be more preferable than in reality.

Further analysis to track the path of each fish was conducted with Animal Tracker [41] in ImageJ. For this application the video recordings of the fish were converted to 4 fps videos using Matlab (2022a) to minimise computational effort whilst still obtaining precise tracking data. The tracking area was designed with the *Zone Designer* module in the program. The selected area included the entire flume section available to the fish and excluded the turbine and its supports as well as everything outside the flume. This *Zone* file was saved and used for all fish and treatments. The *Tracker* module was then used to filter out all objects that were stationary for the duration of the video with the background subtractor. A gaussian blur filter was added to reduce the likelihood of small particles in the flow being picked up by the tracker. A threshold was established for each video separately to maximise the visibility of the fish whilst minimizing any noise or other source of movement in the video. Post-processing filters to exclude objects too big or too small and to erode and dilate the image were applied to further isolate the fish and improve the tracking. Tracking began after the 30 s allowed for the fish to be released into the flume. After tracking, the results were first checked to ensure the fish had been followed accurately and then saved. The tracking was then verified and repeated if necessary. This was used to calculate paths, distances, and velocities of the fish.

### 2.3 Statistical analysis

All data was statistically analysed with R version 4.2.2 [42]. The data was first inspected for normality of the data and checked with a Shapiro-Wilk test. The data was subsequently modelled with a Generalised Linear Model (GLM) using the MASS package [43], the residuals and overdispersion were then inspected and a Box Cox transformation used when necessary. The GLM used was determined by inspecting the AIC value along with residual distributions. A gaussian GLM with identity link function was used to analyse fish length, mass, total distance swam and differences in time spent in a zone or grouping of zones across treatments. Binomial GLMs were used to analyse how many fish were hit by the turbine (probit link) and how many fish passed by the turbine (cauchit link) Where the data was zero inflated, a Zero-Inflated Negative Binomial (ZINB) or a Zero-Inflated Poisson (ZIP) model was used with the pscl package [44]. ZINB models with a logit link function were used to analyse number of passes, rejection, entrain, upstream and comparison of time spent in different zones within the same treatment. A ZIP model with logit link was used for the approach data. For all tests the level of significance used was *p* < 0.05.

## 3 Results

### 3.1 General behaviour and spatial distribution

**Fig. 3:**
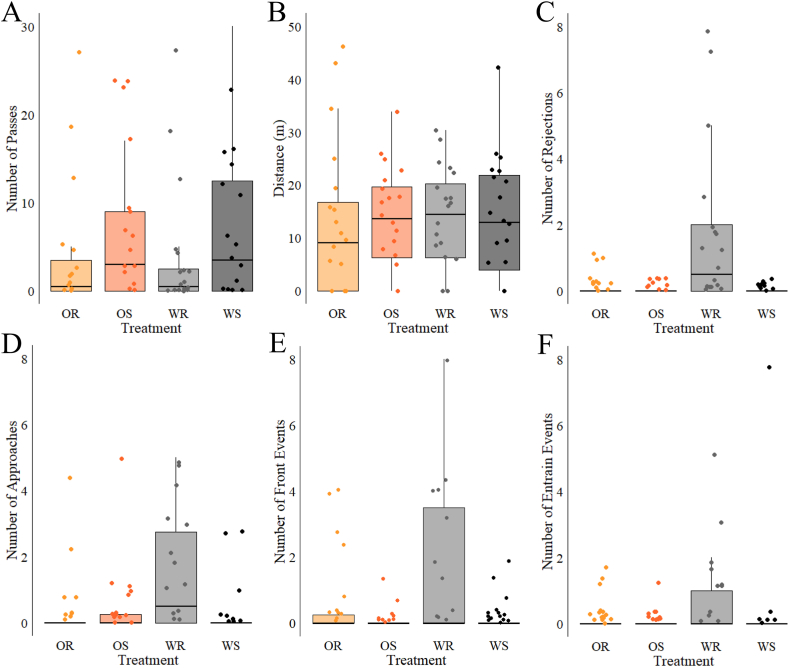
A panel of boxplots of the main behavioural and spatial results, the boxes show interquartile range and the whiskers represent 95 % range of the data. OR = Orange Rotating, OS = Orange Stationary, WR = White Rotating, WS = White Stationary. The plots in this figure show different metrics by treatment, each plot is labelled A–F: A = Passes, B = distance swam, C = number of rejections, D = number of approaches, E = number of bow-waking occurrences, F = number of occurrences of entrainment behind the turbine. (For interpretation of the references to colour in this figure legend, the reader is referred to the Web version of this article.)

Fish approached the turbine significantly more in the white rotating (WR) treatment compared with both orange rotating (OR) and orange stationary (OS) (ZINB, *p* < 0.02, Fig. 3d) treatments. Under the white stationary (WS) treatment there were fewer approaches than WR but not significantly (ZINB, *p* = 0.1).

Fish sharply rejected the turbine significantly more under the WR treatment (GLM, *p* = 0.0002, Fig. 3c), with this behaviour never occurring with the stationary conditions regardless of colour and only to a lesser extent in OR. Physical contact with the turbine was most common in the WR treatment, with five strikes observed compared to one strike for OR and OS and no strikes in treatment WS. The number of strikes was not significant between any treatment (GLM, *p* = 0.9), and the fish experienced no apparent visible damage.

The entrain and bow-waking behaviours, where the fish swam immediately downstream (in the near wake) and immediately upstream of the turbine respectively, were observed most in the WR treatment (Fig. 3e–f). Each time a fish would present these behaviours it was counted as one occurrence. Fish entrained significantly more often in WR than OS (GLM, *p* < 0.01) but not for the other two treatments, although both WS and OR had 21 and 25 fewer occurrences, respectively.

Swimming directly upstream of the turbine was also most frequently observed in WR (Fig. 3e), with a significant difference between treatment WR and both stationary turbine treatments (GLM, *p* < 0.05). Interestingly, OR was also higher than OS (GLM, *p* < 0.0005). This result indicates potential hydrodynamic benefits as the turbine would not be in sight of the fish swimming immediately upstream of the turbine with the turbine directly behind them which means that visual impacts can be reduced in this case.

The number of fish passing the turbine at least once was highest in the stationary treatments, with 12 and 14 fish passing for the white (WS) and orange (OS) configurations respectively (Fig. 3a). In the rotating turbine treatments (WR and OR), only 10 fish passed the turbine at least once but there was overall no significant difference between number of fish to pass upstream of the turbine between any two treatments (GLM, *p* > 0.2). Similarly, the number of passes per fish was highest for WS (9.4 mean passes) and OS (8.4 mean passes) whereas OR and WR had 6 and 4.7 mean passes respectively although no significant difference was found between treatments (ZINB, *p* > 0.3).

**Fig. 4:**
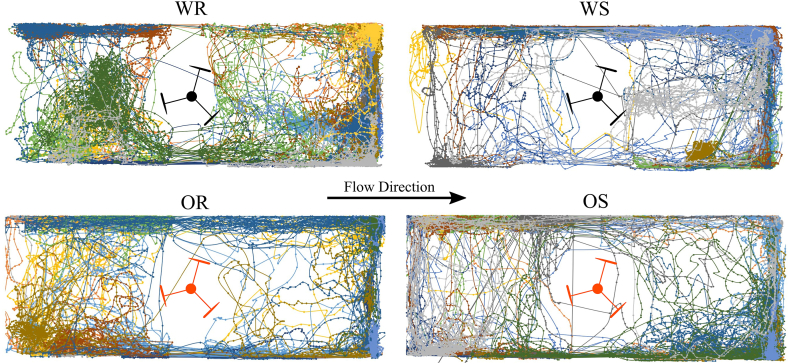
Tracked paths of each individual fish in each treatment computed with Animal Tracker. Each fish's path in each treatment is represented by a different colour and each dot on the tracked line represents each tracking frame (every 0.25 s) for this fish. The paths in the area near the turbine are interpolated since the support structure of the turbine obscured this region. The turbine is not to scale in its internal proportions. Flow from left to right. OR = Orange Rotating, OS = Orange Stationary, WR = White Rotating, WS = White Stationary. (For interpretation of the references to colour in this figure legend, the reader is referred to the Web version of this article.)

**Fig. 5:**
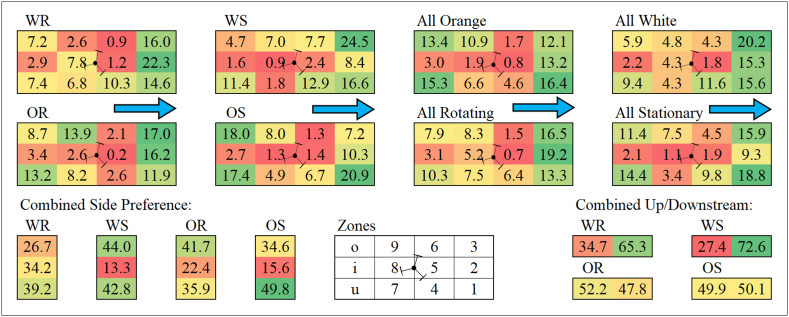
Average percentage of time spent by fish in each zone of the working area. The blue arrows indicate the flow direction, each panel within the figure represents a single treatment or a combination of treatments or zones. The cells within the panels represent a single zone of the working area and are coloured by time spent on a sliding scale from the most time (green) to the least (red). OR = Orange Rotating, OS = Orange Stationary, WR = White Rotating, WS = White Stationary. (For interpretation of the references to colour in this figure legend, the reader is referred to the Web version of this article.)

The trajectories of fish plotted in Fig. 4 revealed that some individuals had spatial preference for one side of the flume or a specific area (e.g. the downstream right hand side next to the wall) while others explored the entire area, highlighting how individual fish behaviour with treatment. For all treatments, there are fewer trajectories within 2 fish lengths of the turbine, which indicates avoidance behaviour. In treatments OS and OR in particular, fewer fish went near the turbine (Fig. 5) and did not repeatedly approach the turbine when compared to treatments WS and WR. This shows that turbine rotation is not solely responsible for fish not approaching the turbine. The lack of trajectories also shows that the fish did not often swim directly in the wake of the turbine did not spend time in areas that would allow them to exploit any hydrodynamic benefits such as the lower velocities. This is further evidenced by the comparatively small proportion of time spent directly in the turbine wake as shown in Fig. 5. The trajectory results also highlight how individual fish behaviour varies within a treatment.

The total distance travelled by fish in the different treatments do not vary significantly (GLM, *p* > 0.8; Fig. 3b). Fish that did not rest for prolonged periods of time against the downstream flow straightener covered around 15 m on average throughout the 10 min experiment.

### 3.2 Temporal distribution

For the analysis of the time spent in each zone of the test section, the zones are referred to by their code specified in Fig. 1 for simplicity, are presented in Fig. 5 along with the temporal distribution. Fish spent significantly more time in the near wake of the turbine (zone 5) in WR than in OR (GLM, *p* < 0.05) and more time in zone 6 in WS than all other treatments (GLM, *p* < 0.05). There was no other significant difference between time spent in other individual zones and between different treatments. The combined time spent in zones 4, 5 and 6 (zones immediately downstream of turbine), however, was significantly more in WS than OR and OS (GLM, *p* < 0.05). Overall, in each treatment the least amount of time was spent in the immediate vicinity of the turbine, particularly zone 5 was avoided compared to other zones (GLM, *p* < 0.05). Zones 1, 2 and 3 (furthest downstream area) were generally used the most but that did not necessarily indicate that more time was spent downstream of the turbine. The areas in the immediate vicinity of the turbine were avoided the most but zones o, i and u (furthest upstream area) were used by the fish but not as much as zone 1, 2 and 3. In treatments OR and OS, combined time spent upstream of the turbine was approximately equal to that spent downstream of the turbine, whereas for WR and WS more time was spent downstream. There was no significant difference in side preference of the fish between treatments, indicating that the asymmetrical wake of the turbine did not cause the fish to prefer one side to the other.

## 4 Discussion

The current study indicates that rainbow trout in the presence of an orange turbine spent less time in the vicinity of the turbine and experienced decreased interactions with it compared to fish encountering a white turbine. Turbine colour did not impact trout passage or general mobility such as distance covered. There was an adverse effect on temporal distribution and behaviour of the trout in the presence of white rotating (WR) turbine; fish in this treatment were most prone to dangerous interactions with the turbine. The orange turbine decreased the risk of fish colliding with the rotating turbine, coming close to it or spending time near it (zones 4, 5 and 6). This is desirable since it does not further fragment their habitat, and connectivity is unchanged but with a decreased chance of the trout being affected by the turbine, even in a channel where the turbine occupies a significant proportion of available space, which can be the case in a river setting.

There are two possible explanations for the observed change in fish behaviour in the presence of differently coloured turbines. Firstly, fish can react differently to specific colours and they may be displaying avoidance behaviour when encountering specific colours. Rainbow trout do react to colour in diverse ways; in particular, the red spectrum negatively affects growth [45,46] and elevates stress levels [46]. Furthermore, an orange coloured leaky barrier increased trout passage [31] and strobes of different colours (including red and orange) are an effective guidance tool for white sturgeon (*Acipenser transmontanus*), walleye (*Sander vitreus*) and European eels (*Anguilla anguilla*) [[33], [34], [35]]. The other explanation for the change in behaviour is simply that the orange turbine is more visible than the white turbine to the trout, either due to the wavelength of the colour or because the orange turbine presented a greater contrast against the background in the flume. The latter seems less likely because the light levels in all treatments were high such that everything in the flume should have been visible to the fish. Despite the uncertainty of the underlying mechanism for the increased visibility of the turbine, higher visibility would allow the trout to detect the turbine at a greater distance, thereby encouraging avoidance movements. The increased visibility explanation is supported by the findings from a wind turbine study where less turbine related fatalities in birds occurred when one of the turbine blades was painted black [30]. The idea that visibility is the main factor influencing fish behaviour is also supported by our current results under the stationary turbine treatments. Here, colour did not cause significantly different results and the overall fish behaviour was similar (Fig. 3). This implies that the stationary turbine was easily visible to the trout as they did not often display evasive behaviour such as rejections upon encountering the stationary turbine. This suggests that the trout were not dissuaded by the turbine when it was not moving, a similar finding to that of Bender et al. (2023) with Atlantic salmon interactions with a VAT. When the white turbine was rotating, the fish swam towards it and rejected it more often. An explanation for this is that they were not directly approaching the turbine because of being attracted to it, instead, the fish were swimming in the middle of the flume and only detected the turbine when close to it which led more frequently to the fish rejecting or colliding with the turbine. Increased rejections and time spent near the turbine may not only result in increased blade contacts, but also implies a waste of energy. When the orange turbine was rotating (OR), the data suggests it was more visible and the trout were able to avoid the turbine as they could detect its position in the flume and swim around it without being surprised and taking sudden evasive action. Importantly, the ‘approach behaviour’ cannot be assumed to indicate fish attraction to the turbine. As the turbine occupies 40 % of the cross-sectional area of the flume, random swimming may explain proximity to the turbine.

The hydrodynamics produced by turbines and how fish react to them should be explored in future studies to explain the observed differences between rotating and stationary conditions. When the turbine is rotating it produces a region of lower velocity immediately downstream of the turbine, with this region also being highly turbulent compared to the rest of the flume [23,47]. A wake is also generated behind the stationary turbine, although smaller in comparison to the rotating turbine's wake. There is also a small region of reduced flow velocity immediately upstream of the turbine (bow wake) but this area was not often used by the fish (Fig. 5). The trout did not spend significantly more time in the wake of the turbine compared to other areas in any of the treatments and it is unclear to what extent the turbulence and/or reduced flow velocity in the wake affected fish motion and behaviour. A potential consequence of the turbine wake, however, is that in confined channels such as this where the velocity on either side of the turbine is increased, turbines can affect passage and fish avoid the turbine wake [28]. That means that in the current experiment the fish were more likely to interact with the turbine considering its size relative to flume width. When discussing the role that vision has in sensing a turbine, the ability of the fish to sense hydrodynamic parameters is also a factor to be considered. Fish predominantly detect flow characteristics using their lateral line organs which detect pressure changes and velocity gradients in the surrounding flow through the mechanosensory hair cells on the neuromasts [48,49]. When reacting to any flow field, a combination of vision and lateral line sensors are responsible for fish behaviour and kinematics [15]. This may explain the differences in behaviour between stationary and rotating treatments in the current study. However, in line with our previous study [28], the turbine wake did not seem to significantly affect fish behaviour in any of the treatments and the behavioural differences observed between treatment WR and the stationary treatments (WS and OS) were also observed when comparing OR and WR. This suggests that the colour change and not the hydrodynamics were responsible for the differences in this experiment.

A further confounding factor when considering field applications is the effect of turbidity since river water is rarely as clear as that used in this experiment. In turbid conditions there would be reduced visibility of the turbine in general and as evidenced by the results of this experiment, the trout used sight when interacting with the turbine. More studies are needed to address the role of turbidity and of light colour, type, and intensity on fish behaviour around turbines. In addition, a factor not considered in this study, but one important to investigate, was latency to pass the turbine. This metric would have been of limited use here considering the relatively short duration of the experiment but in natural conditions the fish may need to navigate multiple barriers so multiple delays could be compounded to negatively affect the fish, as barriers are pervasive in many rivers [3,50]. Lastly, it is important to note that this study is species-specific, rainbow trout have well-developed vision and have known sensitivity to the specific colour used in this study and it is therefore necessary to evaluate other species independently before colour is adopted as a solution.

## 5 Conclusion

In summary, this experiment supports the argument that turbine colour increases visibility and in turn, reduces the threat they pose to aquatic wildlife. This is achieved by alerting trout of the turbine presence, providing more time to select an avoidance pathway. More evidence is needed to fully understand the effect that increased visibility and colour may have on passage and behaviour and which colours most enhance visibility of the turbine and whether this is species dependent. Orange compared to white turbine blades decreased trout interactions with the turbine whilst not significantly affecting the ability of the trout to pass the turbine or swim freely through the working area. Therefore, modifying the appearance of the turbine has the potential to be an effective and low-cost solution to reduce turbine collisions and benefit fish welfare.

## Data Availability

The raw and processed data required to reproduce the above findings are available to download from https://doi.org/10.17632/58vss8ky7r.1.

## CRediT authorship contribution statement

**Guglielmo Sonnino Sorisio:** Conceptualization, Data curation, Formal analysis, Investigation, Methodology, Validation, Visualization, Writing – original draft, Writing – review & editing. **Stephanie Müller:** Data curation, Investigation, Methodology, Writing – review & editing. **Catherine A.M.E. Wilson:** Conceptualization, Funding acquisition, Methodology, Supervision, Writing – review & editing. **Pablo Ouro:** Conceptualization, Supervision, Writing – review & editing. **Jo Cable:** Conceptualization, Funding acquisition, Methodology, Supervision, Writing – review & editing.

## Declaration of competing interest

The authors declare that they have no known competing financial interests or personal relationships that could have appeared to influence the work reported in this paper.
